# Racial and socioeconomic disparities in survival among women with advanced-stage ovarian cancer who received systemic therapy

**DOI:** 10.1007/s10552-023-01810-y

**Published:** 2023-10-24

**Authors:** Caretia J. Washington, Shama D. Karanth, Meghann Wheeler, Livingstone Aduse-Poku, Dejana Braithwaite, Tomi F. Akinyemiju

**Affiliations:** 1 Department of Epidemiology, University of Florida College of Public Health and Health Professions, Gainesville, FL USA; 2 University of Florida Health Cancer Center, Gainesville, FL USA; 3 Department of Surgery, College of Medicine, University of Florida, Gainesville, FL USA; 4 Department of Population Health Sciences, Duke University School of Medicine, Durham, NC 27708 USA; 5 Duke Cancer Institute, Duke University School of Medicine, Durham, NC USA

**Keywords:** Gynecologic cancers, Systemic therapy, Cancer disparities, Socioeconomic status

## Abstract

**Purpose:**

The purpose of this study was to assess the association between race/ethnicity and all-cause mortality among women with advanced-stage ovarian cancer who received systemic therapy.

**Methods:**

We analyzed data from the National Cancer Database on women diagnosed with advanced-stage ovarian cancer from 2004 to 2015 who received systemic therapy. Race/ethnicity was categorized as Non-Hispanic (NH) White, NH-Black, Hispanic, NH-Asian/Pacific Islander, and Other. Income and education were combined to form a composite measure of socioeconomic status (SES) and categorized into low-, mid-, and high-SES. Multivariable Cox proportional hazards models were used to assess whether race/ethnicity was associated with the risk of death after adjusting for sociodemographic, clinical, and treatment factors. Additionally, subgroup analyses were conducted by SES, age, and surgery receipt.

**Results:**

The study population comprised 53,367 women (52.4% ages ≥ 65 years, 82% NH-White, 8.7% NH-Black, 5.7% Hispanic, and 2.7% NH-Asian/Pacific Islander) in the analysis. After adjusting for covariates, the NH-Black race was associated with a higher risk of death versus NH-White race (aHR: 1.12; 95% CI: 1.07,1.18), while Hispanic ethnicity was associated with a lower risk of death compared to NH-White women (aHR: 0.87; 95% CI: 0.80, 0.95). Furthermore, NH-Black women versus NH-White women had an increased risk of mortality among those with low-SES characteristics (aHR:1.12; 95% CI:1.03–1.22) and mid-SES groups (aHR: 1.13; 95% CI:1.05–1.21).

**Conclusions:**

Among women with advanced-stage ovarian cancer who received systemic therapy, NH-Black women experienced poorer survival compared to NH-White women. Future studies should be directed to identify drivers of ovarian cancer disparities, particularly racial differences in treatment response and surveillance.

**Supplementary Information:**

The online version contains supplementary material available at 10.1007/s10552-023-01810-y.

## Background

Ovarian cancer is the fifth leading cause of cancer-related deaths in women in the United States and is estimated to account for 13,270 deaths in 2023 [1]. Efficient screening techniques or simple diagnostic tests for ovarian cancer are currently lacking [2, 3]. Thus, most women with ovarian cancer are diagnosed at advanced stages, leading to poor 5-year survival rates: 49% for White women and 41% for Black women [1, 4]. Given these dismal survival rates, new treatment regimes, including the use of primary systemic therapy, are emerging [5, 6].

Guideline-adherent treatment for advanced-stage ovarian cancer usually involves cytoreductive surgery followed by adjuvant chemotherapy and sometimes radiotherapy [5, 6]. However, many women with advanced ovarian cancer have a high risk of surgical complications and recurrence, and primary systemic therapy has been used for this select group of women [7, 8]. For instance, neoadjuvant chemotherapy, such as carboplatin and paclitaxel, is recommended if there is a low likelihood of achieving optimal primary cytoreductive surgery for women diagnosed with stage III or IV ovarian cancer [7]. Immunotherapy, such as checkpoint inhibitors, has also shown promise for the treatment of recurrent ovarian cancer in recent randomized control trials [8, 9].

Despite the widespread use of systemic therapy, there are racial disparities in survival of advanced-stage ovarian cancer. Several studies have found that Black women experience poorer survival of advanced-stage ovarian cancer compared to their White counterparts [10, 11]. Socioeconomic and access-to-healthcare factors further widen the racial disparity in ovarian cancer [12–14]. Although the inadequate receipt of guideline-recommended treatment has been well documented for its contribution to the racial disparities in ovarian cancer survival, [13, 15–17] the factors that contribute to these disparities among women who have received equivalent treatment are not well understood [18]. Therefore, there is a critical need to evaluate disparities in advanced-stage ovarian cancer survival among women who have received systemic therapy in the United States (U.S.).

In this study, we utilized data from the National Cancer Database (NCDB) to evaluate the association between race/ethnicity and risk of all-cause death among women with advanced-stage ovarian cancer who have equal utilization of systemic therapy. We also investigated whether demographic characteristics, comorbidity level, and receipt of surgery modified those associations.

## Methods

### Data source

The data for this study were obtained from the 2016 NCDB Participant User Files (PUF); the NCDB is a joint program of the American College of Surgeons Commission on Cancer and the American Cancer Society [19]. The NCDB includes over 70% of all patients with newly diagnosed cancers in the United States annually [19]. The data are abstracted by certified tumor registrars as part of the National Cancer Registrars Association [20]. The data includes patient demographics, tumor characteristics, clinical and pathological TNM stage classification, treatments, and survival data. This analysis was considered exempt by the University of Florida Institutional Review Board as the data used in this study were obtained from a de-identified NCDB database.

### Study cohort

The study cohort included women that met the following criteria: (1) stage III-IV ovarian cancer (International Classification of Diseases for Oncology, Third Edition topography code- C56.9), (2) women diagnosed between 2004 and 2015, (3) received systemic therapy (defined as chemotherapy, immunotherapy, and/or hormone therapy by the NCDB), and (4) had no missing values for sociodemographic characteristics, treatment receipt, tumor characteristics, and time elapsed between the date of diagnosis and the date of last contact or death.

### Exposure, outcome, and covariates

The main exposure for this data analysis was self-reported race/ethnicity retrieved from the medical records. The race and ethnicity variables were combined and were categorized into the following groups: non-Hispanic White (NH-White), non-Hispanic Black (NH-Black), Hispanic, non-Hispanic Asian/Pacific Islander (NH-Asian/PI), and Other (Hawaiian, Micronesian, Chamorran, Guamanian, Polynesian, Tahitian, Samoan, Tongan, Melanesian, Fiji Islander, and New Guinean). Hereafter, we exclude the NH prefix when referencing racial groups. Our outcome of interest was all-cause mortality, with the survival time measured (in months) from the date of diagnosis to death or last contact (whichever occurred first). The sociodemographic covariates included in this study were age at diagnosis (< 65 and ≥ 65 years), area-level educational level as the percentage of individuals in the patient’s zip code without a high school degree categorized into quartiles based on all United States (US) zip codes (21% or more, 13-20.9%, 7-12.9%, and < 7%), and median household income was estimated by zip code of the patient recorded at the time of diagnosis and categorized as quartiles (< $38,000, 2: $38,000 -$47,999, $48,000-$62,999, and ≥ $63,000) [21]. The quartile classifications for area-level educational level and median household income were combined to form a composite score for SES groups: low (2–3), mid (4–7), and high (8) (Supplementary Table 1) [22]. Healthcare access factors included primary insurance (no insurance, private insurance/managed care, and government insurance) and cancer treatment facility type (academic and non-academic). Treatment receipt was defined as receipt of chemotherapy (yes vs. no), receipt of immunotherapy (yes vs. no), receipt of hormone therapy (yes vs. no), receipt of surgery (yes vs. no), and receipt of radiation therapy (yes vs. no). Charlson-Deyo comorbidity index (CCI) score was categorized as no comorbidities and one or more comorbidities. Additional covariates included tumor grade (low, intermediate, high, and unknown) histology (high-grade serous carcinoma, low-grade serous, serous NOS, clear cell, endometroid, mucinous, mixed, and other) [23]. Covariates were selected based on *a priori* knowledge regarding their associations with the exposure and outcome of interest.

### Statistical analysis

The distributions of study covariates were summarized in the overall sample and by race/ethnicity. Kaplan-Meier curves were used to visualize the probability of survival by race/ethnicity overall and by each SES group, and the differences in survival probabilities were examined by the log-rank test. Number of deaths and mortality rates (reported as the number of deaths per 10 person-years) were summarized for the overall study population and according to race/ethnicity and further by stratified age, CCI, SES, and surgery receipt. The association between racial/ethnic groups (reference: White) and risk of all-cause mortality was examined in three multivariable Cox proportional hazards models. The first model was adjusted for age and SES; the second model was additionally adjusted for primary insurance and cancer facility type; and the final model was additionally adjusted for CCI, treatment receipt, histology, and grade. In the final model, all covariates (age, SES, facility type, primary insurance, radiation receipt, surgery receipt, cancer histology, grade, and CCI) violated the proportional hazard assumptions. Therefore, we created and controlled for the interaction terms between the covariates and follow-up time, and no violation was observed afterward.

Subgroup analyses were conducted by age at diagnosis, CCI, and SES. The interaction term between race/ethnicity and age at diagnosis, CCI, or SES was added into the multivariable Cox proportional hazards models. Subgroup analyses were also conducted based on surgery receipt. The significance of interaction tests was determined by the Wald test. All analyses were performed using SAS 9.4 (Cary, NC) and R studio V 4.1.1. All statistical tests were 2-sided, and a p-value < 0.05 indicated statistical significance.

## Results

The final study sample included 53,367 women diagnosed with advanced-stage ovarian cancer and had received systemic therapy (Supplementary Fig. 1). Table 1 presents the demographic and clinical characteristics of women diagnosed with advanced-stage (III-IV) primary ovarian cancer from 2004 to 2015. Approximately 50% of the women were ≥ 65 years old at the time of diagnosis, the majority did not have any comorbidities (78.0%), and most were White (82.0%), followed by Black (8.7%), Hispanic (5.7%), Asian/Pacific Islander (2.7%), and Other (0.9%). Most women had government insurance (55.8%) and lived in regions classified as the mid-SES group (61.1%). White women (22.3%) made up the largest proportion of women living in regions classified as the high-SES group. Women who identified as Black, Hispanic, Asian/PI, or Other were more likely to be younger (< 65 years) at diagnosis and more likely to be treated at academic facilities. Additionally, Black women were less likely to receive surgery (64.0% Black vs. 73.9% White) and more likely to have rarer histological types of ovarian cancer (40.2% Black vs. 34.8% White). The median time from diagnosis to last follow-up or death was 21.7 months for White women and 19.5 months for Black women (Table 1).

**Table 1:** Sociodemographic and Cancer Characteristics for Advanced-Stage (III-IV) Ovarian Cancer Patients Who Received Systemic Therapy

	Overall (%)					Race n (%)				
	N = 53,367		NH-White N = 43,760 (82.0)		NH-Black N = 4634 (8.7)	Hispanic N = 3062 (5.7)		NH-Asian/PI N = 1449 (2.7)		Other N = 462 (0.9)
**Age**										
< 65	25,411 (47.6)		19,855 (45.4)		2536 (54.7)	1831 (59.8)		911 (62.9)		278 (60.2)
65+	27,956 (52.4)		23,905 (54.6)		2098 (45.3)	1231 (40.2)		538 (37.1)		184 (39.8)
**Charlson-Deyo Comorbidity score**										
0	41,621 (78.0)		34,528 (78.9)		3198 (69.0)	2364 (77.2)		1185 (81.8)		346 (74.9)
1	11,746 (22.0)		9232 (21.1)		1436 (31.0)	698 (22.8)		264 (18.2)		116 (25.1)
**Primary insurance**										
Not insured	1977 (3.7)		1182 (2.7)		278 (6.0)	378 (12.3)		109 (7.5)		30 (6.5)
Private	21,608 (40.5)		17,942 (41.0)		1682 (36.3)	1081 (35.3)		726 (50.1)		177 (38.3)
Government	29,782 (55.8)		24,636 (56.3)		2674 (57.7)	1603 (52.4)		614 (42.4)		255 (55.2)
**SES Composite Groups**										
Low SES	9962 (18.7)		6301 (14.4)		2153 (46.5)	1183 (38.6)		191 (13.2)		134 (29.0)
Mid SES	32,588 (61.1)		27,694 (63.3)		2166 (46.7)	1606 (52.5)		873 (60.3)		249 (53.9)
High SES	10,817 (20.3)		9765 (22.3)		315 (6.8)	273 (8.9)		385 (26.6)		79 (17.1)
**Cancer facility type**										
Academic	21,916 (41.1)		17,135 (39.2)		2273 (49.1)	1516 (49.5)		753 (52.0)		239 (51.7)
Not academic	31,451 (58.9)		26,625 (60.8)		2361 (51.0)	1546 (50.5)		696 (48.0)		223 (48.3)
**Chemotherapy**										
Yes	53,171 (99.6)		43,591 (99.6)		4620 (99.7)	3057 (99.8)		1446 (99.8)		457 (98.9)
No	196 (0.4)		169 (0.4)		14 (0.3)	5 (0.2)		3 (0.2)		5 (1.1)
**Immunotherapy**										
Yes	804 (1.5)		671 (1.5)		64 (1.4)	48 (1.6)		18 (1.2)		3 (0.7)
No	52,563 (98.5)		43,089 (98.5)		4570 (98.6)	3014 (98.4)		1431 (98.8)		459 (99.4)
**Hormone therapy**										
Yes	834 (1.6)		677 (1.6)		72 (1.6)	51 (1.7)		23 (1.6)		11 (2.4)
No	52,533 (98.4)		43,083 (98.5)		4562 (98.5)	3011 (98.3)		1426 (98.4)		451 (97.6)
**Surgery Receipt**										
Yes	39,042 (73.2)		32,323 (73.9)		2966 (64.0)	2261 (73.8)		1147 (79.2)		345 (74.7)
No	14,325 (26.8)		11,437 (26.1)		1668 (36.0)	801 (26.2)		302 (20.8)		117 (25.3)
**Radiation Receipt**										
Yes	682 (1.3)		547 (1.3)		70 (1.5)	40 (1.3)		23 (1.6)		2 (0.4)
No	52,685 (98.7)		43,213 (98.8)		4564 (98.5)	3022 (98.7)		1426 (98.4)		460 (99.6)
**Grade**										
Low	956 (1.8)		792 (1.8)		74 (1.6)	57 (1.9)		27 (1.9)		6 (1.3)
Intermediate	3327 (6.2)		2781 (6.4)		263 (5.7)	175 (5.7)		87 (6.0)		21 (4.6)
High	29,594 (55.5)		24,424 (55.8)		2346 (50.6)	1681 (54.9)		856 (59.1)		287 (62.1)
Unknown	19,490 (36.5)		15,763 (36.0)		1951 (42.1)	1149 (37.5)		479 (33.1)		148 (32.0)
**Histology**										
H/G Serous	3098 (5.8)		2653 (6.1)		207 (4.5)	146 (4.8)		66 (4.6)		26 (5.6)
L/G Serous	10,647 (20.0)		8809 (20.1)		916 (19.8)	598 (19.5)		243 (16.8)		81 (17.5)
Serous NOS	18,959 (35.5)		15,679 (35.8)		1470 (31.7)	1068 (34.9)		568 (39.2)		174 (37.7)
Clear cell	1159 (2.2)		921 (2.1)		81 (1.8)	67 (2.2)		77 (5.3)		13 (2.8)
Endometrioid	1351 (2.5)		1087 (2.5)		103 (2.2)	104 (3.4)		47 (3.2)		10 (2.2)
Mucinous	919 (1.7)		696 (1.6)		123 (2.7)	68 (2.2)		25 (1.73)		7 (1.5)
Mixed	1643 (3.1)		1381 (3.2)		110 (2.4)	91 (3.0)		46 (3.2)		15 (3.3)
Other/NOS	15,591 (29.2)		12,534 (28.6)		1624 (35.1)	920 (30.1)		377 (26.0)		136 (29.4)
**Stage**										
3	30,278 (56.7)		25,179 (57.5)		2346 (50.6)	1669 (54.5)		822 (56.7)		262 (56.7)
4	23,089 (43.3)		18,581 (42.5)		2288 (49.4)	1393 (45.5)		627 (43.3)		200 (43.3)

Column percentages were reported in the table.

Abbreviations: NH, Non-Hispanic; PI, Pacific Islander; AI, American Indian; AN, Alaskan Native; NOS, Not Otherwise Specified;

H/G, High grade; L/G, Low Grade, SES, Socioeconomic status (education and income).

**Fig. 1:**
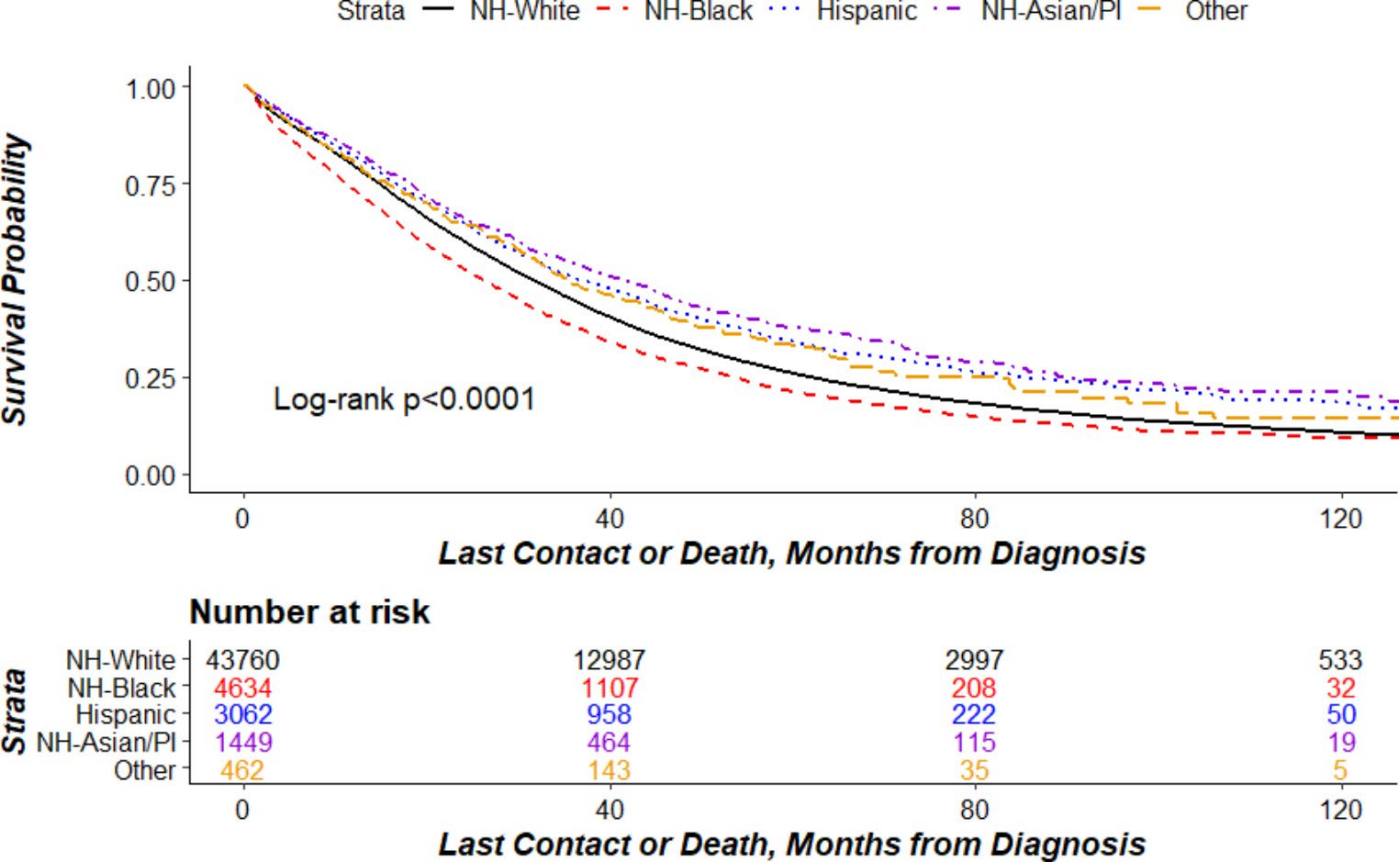
Kaplan Meir curve of Patients with Advanced-Stage Ovarian Cancer with Systemic Therapy Receipt by Race/Ethnicity. Abbreviations: NH non-Hispanic. PI Pacific Islander.

Kaplan-Meier curves by race/ethnicity (Fig. 1) indicated the lowest survival probabilities among Black women (log-rank *P* < 0.0001).

**Fig. 2:**
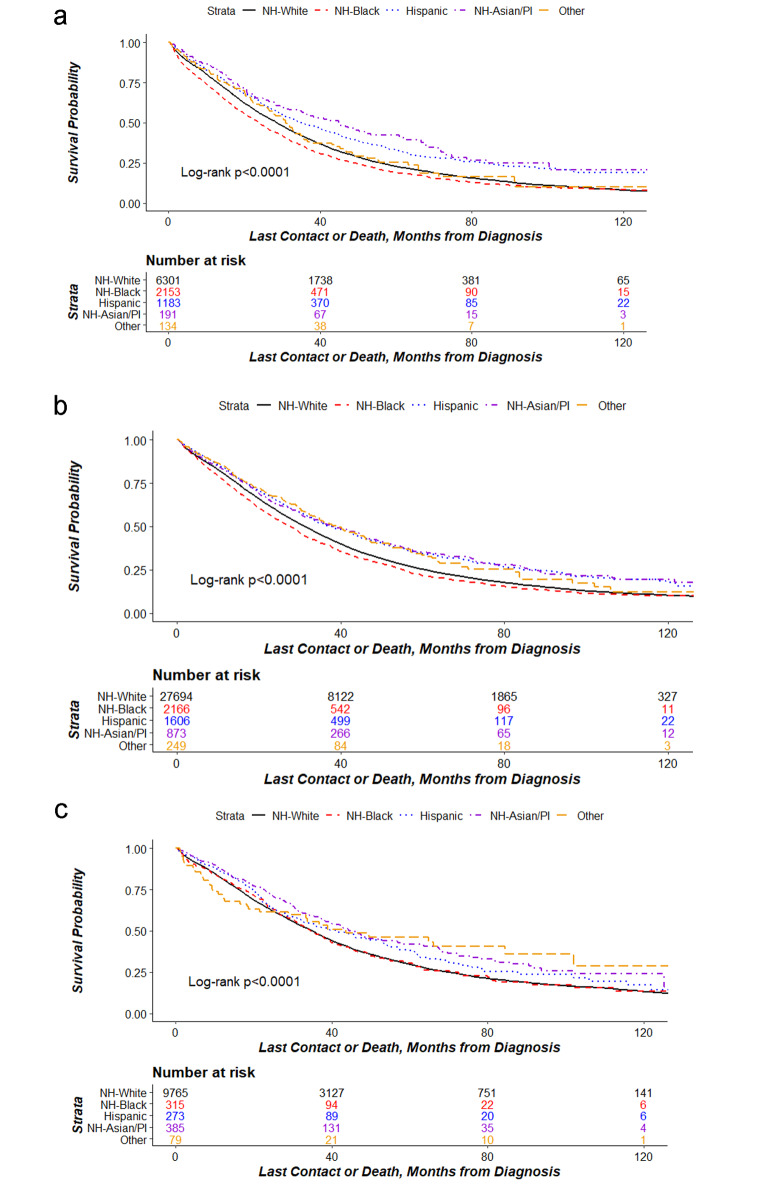
Kaplan Meir curves of Patients with Advanced-Stage Ovarian Cancer with Systemic Therapy by Socioeconomic Categories. (**a**). Low SES Group. Kaplan Meier curves for survival probability by race among the low socioeconomic group. Abbreviations: NH indicates non-Hispanic. (**b**). Mid SES Group. Kaplan Meier curves for survival probability by race among the middle socioeconomic group. NH indicates non-Hispanic. (**c**). High SES Group. Kaplan Meier curves for survival probability by race among the high socioeconomic group. NH indicates non-Hispanic.

Furthermore, Kaplan-Meier curves, stratified by SES, show lower survival probabilities among Black women from both low- and mid-SES groups but not the high-SES group (Fig. 2).

The death rate for Black women with ovarian cancer was 3.06 deaths/10 person-years: 1.19 times the death rate for White women (Table 2). In the Cox proportional hazards regression models assessing all-cause mortality, Black women had a higher risk of death compared to White women across all three adjusted models [fully adjusted hazard ratio (aHR): 1.12; 95% CI: 1.07,1.18)], while Hispanic women had a lower risk of death compared to White women (aHR: 0.87; 95% CI: 0.80,0.95) (Table 2).

Tests of interaction for age and CCI were non-significant across all the fully adjusted models. The tests of interaction for surgery receipt were not significant for the first model (*P*-interaction = 0.07) neither for the fully adjusted model (*P*-interaction = 0.20) (Supplementary Table 2). However, in fully adjusted models stratified by age, a significant association persisted among Black women (< 65 years, aHR: 1.13; 95% CI: 1.05–1.22; ≥65 years aHR: 1.09; 95% CI: 1.02–1.17), while Hispanic women continued to have a lower risk of death relative to White women (< 65 years, aHR: 0.87; 95% CI: 0.77–0.99; ≥65 years aHR: 0.87; 95% CI: 0.78–0.98), (Table 2). In fully adjusted Cox proportional hazards models stratified by SES groups (Table 2), Black women experienced increased risk of mortality compared to White women in the low-SES group (aHR:1.12; 95% CI:1.03–1.22) and mid-SES group (aHR: 1.13; 95% CI:1.05–1.21); while Hispanic women had lower risk of mortality compared to White women in the both the low- and mid-SES groups. Tests of interaction for SES and race/ethnicity were non-significant across all models.

**Table 2:** Cox Proportional Hazards Models of All-cause Death, Stratified by Race, Ethnicity, Age, Comorbidity Score, and Socioeconomic status

					HR and 95% CI		
Race/Ethnicity	**n/N**	**Person-years**	**Death Rate/10 person-years**	Model 1 *	**Model 2†**	**Model 3‡**	
**Overall**							
NH-White NH-Black Hispanic NH-Asian/PI Other	30,248/43,760 3295/4634 1771/3062 776/1449 279/462	117,930 10,759 8515 4049 1260	2.57 (2.54, 2.59) 3.06 (2.96, 3.17) 2.08 (1.99, 2.18) 1.92 (1.79, 2.06) 2.21 (1.97, 2.49)	REF **1.29 (1.22, 1.35)** 0.98 (0.90, 1.06) 1.04 (0.92, 1.17) **1.33 (1.09, 1.62)**	REF **1.30 (1.24, 1.37)** 0.99 (0.91, 1.07) 1.08 (0.95, 1.20) **1.40 (1.15, 1.70)**	REF **1.12 (1.07. 1.18)** **0.87 (0.80, 0.95)** 0.94 (0.83, 1.07) 1.11 (0.91, 1.37)	
Age < 65							
NH-White NH-Black Hispanic NH-Asian/PI Other	12,445/19,855 1684/2536 961/1,831 450/911 154/278	61,132 6556 5565 2702 853	2.04 (2.00, 2.07) 2.57 (2.45, 2.69) 1.73 (1.62, 1.84) 1.67 (1.52, 1.83) 1.81 (1.54, 2.11)	REF **1.31 (1.22, 1.41)** 0.98 (0.86, 1.10) 1.09 (0.91, 1.31) **1.37 (1.02, 1.84)**	REF **1.27 (1.18, 1.36)** 0.94 (0.83, 1.06) 1.11 (0.93, 1.33) **1.40 (1.04, 1.88)**	REF **1.13 (1.05, 1.22)** **0.87 (0.77, 0.99)** 1.02 (0.84, 1.22) 1.20 (0.88, 1.63)	
Age ≥ 65							
NH-White NH-Black Hispanic NH-Asian/PI Other	17,803/23,905 1611/2098 810/1231 326/538 125/184	56,798 4203 2950 1348 407	3.13 (3.09, 3.18) 3.83 (3.65, 4.02) 2.75 (2.56, 2.94) 2.42 (2.17, 2.69) 3.07 (2.57, 3.65)	REF **1.23 (1.15, 1.31)** 0.94 (0.84, 1.05) 0.90 (0.76, 1.07) 1.21 (0.92, 1.60) P-interaction = 0.45	REF **1.27 (1.18, 1.35)** 0.97 (0.87, 1.09) 0.95 (0.80, 1.13) 1.27 (0.96, 1.67) P-interaction = 0.42	REF **1.09 (1.02, 1.17)** **0.87 (0.78, 0.98)** 0.85 (0.71, 1.01) 1.05 (0.79, 1.38) P-interaction = 0.56	
Comorbidity = 0							
NH-White NH-Black Hispanic NH-Asian/PI Other	23,384/34,528 2226/3198 1323/2364 627/1185 198/346	96,227 7798 6844 3366 1013	2.43 (2.40, 2.46) 2.86 (2.74, 2.98) 1.93 (1.83, 2.04) 1.86 (1.72, 2.01) 1.96 (1.70, 2.24)	REF **1.27 (1.20, 1.35)** 0.96 (0.87, 1.06) 1.03 (0.89, 1.19) 1.23 (0.97, 1.56)	REF **1.28 (1.20, 1.35)** 0.96 (0.87, 1.06) 1.08 (0.93, 1.25) **1.29 (1.02, 1.64)**	REF **1.13 (1.07, 1.20)** **0.87 (0.79, 0.96)** 0.95 (0.82, 1.11) 1.06 (0.83, 1.36)	
Comorbidity ≥ 1							
NH-White NH-Black Hispanic NH-Asian/PI Other	6864/9232 1069/1436 448/698 149/264 81/116	21,703 2961 1671 683 247	3.16 (3.09, 3.24) 3.61 (3.40, 3.83) 2.68 (2.44, 2.94) 2.18 (1.85, 2.55) 3.28 (2.62, 4.06)	REF **1.17 (1.07, 1.27)** 0.91 (0.79, 1.07) 0.82 (0.64, 1.05) 1.27 (0.89, 1.81) P-interaction = 0.21	REF **1.19 (1.09, 1.30)** 0.93 (0.80, 1.08) 0.87 (0.69, 1.11) 1.34 (0.94, 1.92) P-interaction = 0.25	REF 1.08 (0.99, 1.18) 0.86 (0.74, 1.00) 0.85 (0.66, 1.084) 1.23 (0.85, 1.76) P-interaction = 0.25	
Low SES							
NH-White NH-Black Hispanic NH-Asian/PI Other	4580/6301 1593/2153 702/1,183 101/191 91/134	16,071 4756 3260 548 341	2.85 (2.77, 2.93) 3.35 (3.19, 3.52) 2.15 (2.00, 2.32) 1.84 (1.51, 2.23) 2.70 (2.16, 3.26)	REF **1.32 (1.21, 1.43)** 0.95 (0.82, 1.10) 0.91 (0.70, 1.20) 1.42 (1.00, 2.02)	REF **1.32 (1.22, 1.44)** 0.96 (0.83, 1.11) 0.94 (0.71, 1.23) **1.48 (1.04, 2.10)**	REF **1.12 (1.03, 1.22)** **0.83 (0.71, 0.96)** 0.81 (0.62, 1.08) 1.10 (0.76, 1.60)	
Mid SES							
NH-White NH-Black Hispanic NH-Asian/PI Other	19,287/27,694 1504/2166 914/1606 484/873 149/249	74,165 5132 4452 2373 701	2.60 (2.56, 2.64) 2.93 (2.79, 3.08) 2.05 (1.92, 2.19) 2.04 (1.86, 2.23) 2.13 (1.80, 2.49)	REF **1.28 (1.19, 1.37)** 0.98 (0.88, 1.10) 1.04 (0.89, 1.22) 1.25 (0.95, 1.64)	REF **1.29 (1.21, 1.38)** 0.98 (0.88, 1.10) 1.08 (0.92, 1.27) **1.32 (1.01, 1.73)**	REF **1.13 (1.05, 1.21)** **0.88 (0.79, 0.99)** 0.94 (0.79, 1.10) 1.08 (0.81, 1.42)	
High SES							
NH-White NH-Black Hispanic NH-Asian/PI Other	6381/9765 198/315 155/273 191/385 39/79	27,694 870 802 1128 218	2.30 (2.25, 2.36) 2.28 (1.98, 2.61) 1.93 (1.65, 2.26) 1.69 (1.47, 1.95) 1.79 (1.29, 2.42)	REF 1.13 (0.96, 1.34) 1.04 (0.82, 1.31) 1.04 (0.77, 1.39) 1.28 (0.76, 2.16) P-interaction = 0.25	REF 1.13 (0.96, 1.33) 1.02 (0.81, 1.30) 1.07 (0.80, 1.44) 1.30 (0.77, 2.19) P-interaction = 0.21	REF 1.07 (0.90, 1.26) 0.97 (0.77, 1.22) 1.05 (0.78, 1.41) 1.18 (0.71, 1.97) P-interaction = 0.61	

Bold value indicates P < 0.05.

*Adjusted for age (not included in models stratified by age) and socioeconomic status.

†In addition, adjusted for insurance, and academic facility type.

‡In addition, adjusted for radiation receipt, surgery receipt, cancer histology, grade, and Charlson/Deyo comorbidity index score (not included in models stratified by Charlson/Deyo score).

CI indicates confidence interval; HR, hazard ratio; n, number of deaths; N, total number of individuals.

Death rate calculated as number of deaths divided by person-years (reported as cases/10-person years).

## Discussion

In the present analysis of women diagnosed with advanced-stage ovarian cancer who received systemic therapy, we observed that the risk of all-cause mortality among Black women was 12% higher than among White women. The increased risk of all-cause mortality observed in Black women spans all age groups and encompasses the low and mid SES categories, further emphasizing the disparity between Black and White women. Conversely, Hispanic women had a lower overall risk of all-cause mortality, across all models, compared to White women. However, there was no evidence of effect modification between race/ethnicity, age, CCI, surgery, and SES groups. These results emphasize the need to address racial disparities in the survival of advanced-stage ovarian cancer, as racial disparities persist even among women who have all received systemic treatment.

Our results are consistent with prior research suggesting that racial disparities in ovarian cancer survival persist, even when all women received systemic treatment [18, 24, 25]. A cohort study among members of Kaiser Permanente Northern California found that Black women experienced poor survival rates compared to White women, even when both groups had equal access to care and received the same systemic therapy (specifically adjuvant first-line therapy of carboplatin and paclitaxel) [18]. The study also reported that Black women were more likely to have rare histological types of ovarian cancer, similar to the findings of our study (grouped in the “Other or NOS histological types” category) [18]. However, even among more rare histological types, such as malignant ovarian germ cell tumors, poorer survival was still found among Black women compared to their White counterparts, despite having similar adjuvant treatment patterns (including surgery, radiation, and chemotherapy) [24]. Another study using NCDB data reported that Black women were more likely to receive neoadjuvant chemotherapy before surgery (which the authors define as the initiation of chemotherapy between 1 and 120 days before interval debulking surgery), as opposed to primary surgery plus adjuvant chemotherapy. This finding may be due to extensive tumor burden or prediction of poor surgical performance, yet Black women still experienced lower survival compared to their White counterparts [26, 27]. Our study’s findings support this conclusion, as we found that the disparity between Black and White women persisted even among women who only received primary surgery. However, our study adds to the existing literature as we found that the racial disparity in advanced-stage ovarian cancer persists despite receipt of systemic therapy. Furthermore, our study adds to the literature showing that the Black-White disparity in ovarian cancer survival persist, even among equivalent chemotherapy receipt [18]. Our study also found that Black women in the low- and mid-SES groups, but not the high SES groups, exhibited poorer survival rates compared to all other racial/ethnic groups. However, Hispanic women within the same SES groups demonstrated better survival rates compared to White women. Similarly, Park et al. found that Black women had poorer survival rates while Hispanic women had better 5-year survival rates across histological types compared to White women [28]. This study, along with other studies on racial disparities in ovarian cancer, is consistent with the ‘Hispanic Paradox’ phenomenon. Despite facing socioeconomic barriers to health, Hispanic individuals have similar or better survival than their White counterparts [29, 30]. Additional studies are needed to evaluate the ethnic differences that might contribute to survival outcomes among women with ovarian cancer [30, 31].

Several clinical differences may also play a significant role in the Black-White disparity in ovarian cancer survival. Our study found that the Black-White disparity persisted even among women who did not report any comorbidities. This finding indicates that healthcare disparities, such as access to and utilization of healthcare resources, as well as other clinical factors, may be at play [32, 33]. For instance, a study from the Ovarian Cancer in Women of African Ancestry consortium found that nulliparity, body mass index, and postmenopausal hormone therapy duration were all independent mediators in the racial disparity in ovarian cancer survival [32]. Biological differences may also contribute to the racial disparity in ovarian cancer, as the highest levels of mutations in micro-RNA (miRNA) genes, which can lead to dysregulation of miRNA processing or degradation, have been found among African American women [34]. Additionally, Black women with ovarian cancer are more likely to have a high expression of immune cells, which is associated with a favorable response to immunotherapy, [35–37] yet they are less likely to be enrolled in clinical trials involving immunotherapy [38]. Therefore, there are most likely clinical and biological factors that remain unaddressed, contributing to the racial disparities in the survival of advanced-stage ovarian cancer, regardless of treatment receipt.

Our study has several strengths. First, it included a large sample size from the NCDB, which undergoes strict quality control measures to ensure high-quality standardized data [19]. Additionally, we were able to adjust for many potential confounders, including demographic, comorbidities, treatment, and tumor characteristics. Furthermore, we were able to make a composite measure of SES to evaluate its role in survival disparities. We comprehensively evaluated the effect modification by age, SES, CCI, and receipt of surgery. However, our study had limitations that need to be considered. First, although the NCDB covers approximately 70% of the U.S. population, the results may not be generalizable to all women with advanced-stage ovarian cancer, as women treated at Commission on Cancer (CoC) accredited facilities may have different sociodemographic characteristics than women treated at non-CoC accredited facilities [19]. Second, the NCDB only records first-course treatments, meaning that patients who receive systemic therapy after their primary treatment were not able to be included in our study nor could we evaluate neoadjuvant versus adjuvant chemotherapy [19]. Furthermore, NCDB reports systemic therapy as chemotherapy, hormone therapy, immunotherapy, or hematologic transplant and endocrine procedures; however, we did not examine hematologic transplant and endocrine procedures nor does the definition specify targeted therapy. Lastly, we were not able to evaluate ovarian cancer-specific death, as the database only records vital status and not the specific causes of death. Despite these limitations, our study allowed us to evaluate disparities in race/ethnicity and SES among women with advanced-stage ovarian cancer who have received systemic therapy while maintaining sufficient power.

## Conclusions

This U.S. clinical registry study evaluated the racial/ethnic and socioeconomic disparities in survival among women with advanced-stage ovarian cancer who have received systemic treatment. Our findings revealed that Black women, compared to White women, experienced poor survival rates despite receiving systemic therapy. This Black-White disparity remains prevalent across all age groups, and in low- and mid-SES backgrounds, aligning with existing literature. Therefore, we urge the development and implementation of multitargeted interventions and policies that address these disparities and strive to reduce and/or eliminate the racial disparity in overall survival among women with advanced-stage ovarian cancer.

### Electronic supplementary material

Below is the link to the electronic supplementary material.

Supplementary Material 1

## Data Availability

The datasets used and/or analyzed during the current study are available from the corresponding author on reasonable request.
