# Supplementary material for: Racial and socioeconomic disparities in survival among women with advanced-stage ovarian cancer who received systemic therapy
Source: Cancer Causes Control. 2023 Oct 24;35(3):487–96. doi: 10.1007/s10552-023-01810-y (PMC10838826; doi:10.1007/s10552-023-01810-y)
Supplement: Supplementary file 1 — Supplementary Material 1 [file 10552_2023_1810_MOESM1_ESM.docx]

Systemic Therapy (2004-2015)

N= 134,415

Figure 1. Flowchart of study participants selection

Excluded n= 3,260

Missing education, income, insurance, and cancer facility

Participants

n= 59,773

Participants

n= 56,627

Excluded n= 3,146

Missing Race

Excluded n= 74,642

- Stage 0,1,2, unknown

Final Analytic Sample

n= 53,367

**eTable1. Distribution of Income and Education and the Composite Socioeconomic Status (SES) Categories**

|  | Total  (N= 53,367) | Low SES  (N= 9,962) | Mid SES  (N= 32,588) | High SES  (N= 10,817) |
| --- | --- | --- | --- | --- |
| **Income** |  |  |  |  |
| 1: <$38,000 | 8,444 (15.8%) | 7,642 (76.7%) | 802 (2.5%) | 0 (0.0%) |
| 2: $38,000-$47,999 | 12,031 (22.5%) | 2,320 (23.3%) | 9,711 (29.8%) | 0 (0.0%) |
| 3: $48,000-$62,999 | 14,413 (27.0%) | 0 (0.0%) | 14,413 (44.2%) | 0 (0.0%) |
| 4: ≥63,000 | 18,479 (34.6%) | 0 (0.0%) | 7,662 (23.5%) | 10,817 (100.0%) |
| **Education** |  |  |  |  |
| 1: ≥21% No HSD | 8,578 (16.1%) | 7,024 (70.5%) | 1,554 (4.8%) | 0 (0.0%) |
| 2: 13-20% No HSD | 13,375 (25.1%) | 2,938 (29.5%) | 10,437 (32.0%) | 0 (0.0%) |
| 3: 7-12.9% No HSD | 17,562 (32.9%) | 0 (0.0%) | 17,562 (53.9%) | 0 (0.0%) |
| 4: <7.0% No HSD | 13,852 (26.0%) | 0 (0.0%) | 3,035 (9.3%) | 10,817 (100.0%) |

The quartile assignments of income and education level were added together to form three composite SES categories, assigned as follows: 2-3=Low SES; 4-7= Mid SES; and 8=High SES. HSD, high school degree.

**eTable2. Cox Proportional Hazards Models Predicting Risk of All-cause Death, Stratified by Race, Ethnicity, and Surgery Receipt**

|  | |  |  |  |  | HR and 95% CI |  |
| --- | --- | --- | --- | --- | --- | --- | --- |
| Race/Ethnicity | | **Crude Death Rate** | **Person-years** | **Death Rate**  **(per 10 person-years)** | **Model 1 *** | **Model 2†** | **Model 3‡** |
| Surgery Receipt=Yes | | | | | | | |
| NH-White  NH-Black  Hispanic  NH-Asian/PI  Other | 20,540/32,323  1,896/2,966  1,182/2,261  555/1,147  186/345 | | 100,7348,458  7,111  3,560  1,116 | 2.04 (2.01, 2.07)  2.24 (2.14, 2.34)  1.66 (1.57, 1.76)  1.56 (1.43, 1.69)  1.67 (1.44, 1.92) | REF  **1.24 (1.15, 1.34)**  1.04 (0.91, 1.19)  1.14 (0.94, 1.39)  **1.50 (1.09, 2.09)** | REF  **1.24 (1.15, 1.34)**  1.05 (0.92, 1.20)  1.19 (0.97, 1.45)  **1.57 (1.14, 2.18)** | REF  **1.22 (1.13, 1.31)**  1.02 (0.89, 1.17)  1.14 (0.94, 1.39)  **1.48 (1.07, 2.06)** |
| Surgery Receipt=No | | | | | | | |
| NH-White  NH-Black  Hispanic  NH-Asian/PI  Other | | 9,708/11,4371,399/1,668  589/801  221/302  93/117 | 17,196  2,300  1,404  489  144 | 5.65 (5.53, 5.76)  6.08 (5.77, 6.41)  4.20 (3.87, 4.54)  4.52 (3.95, 5.15)  6.45 (5.24, 7.88) | REF  1.06 (0.99, 1.14)  **0.77 (0.68, 0.87)**  **0.83 (0.69, 1.00)**  1.07 (0.81, 1.40)  *P*-interaction= 0.07 | REF  1.06 (0.99, 1.14)  **0.76 (0.67, 0.85)**  **0.83 (0.69, 0.99)**  1.05 (0.80, 1.39)  *P*-interaction= 0.11 | REF  1.04 (0.97, 1.11)  **0.75 (0.67, 0.84)**  **0.82 (0.68, 0.99)**  1.03 (0.78, 1.35)  *P*-interaction=0.20 |

Bold value indicates P<0.05.

*Adjusted for age and socioeconomic status.

†In addition, adjusted for insurance, and academic facility type.

‡In addition, adjusted for radiation receipt, cancer histology, grade, and Charlson/Deyo comorbidities score.

CI indicates confidence interval; HR, hazard ratio.

Crude death rate calculated by the number of deaths divided by the total number of individuals.

Death rate calculated as number of deaths divided by person-years (reported as cases/10-person years).

Other race includes Hawaiian, Micronesian, Chamorran, Guamanian, Polynesian, Tahitian, Samoan, Tongan, Melanesian, Fiji Islander, and New Guinean. Abbreviations: NH, Non-Hispanic; PI, Pacific Islander; AI, American Indian; AN, Alaskan Native; NOS, Not Otherwise Specified. SES, Socioeconomic status (education and income).
